# Bolwig Organ and Its Role in the Photoperiodic Response of *Sarcophaga similis* Larvae

**DOI:** 10.3390/insects14020115

**Published:** 2023-01-23

**Authors:** Kazuné Hirata, Sakiko Shiga

**Affiliations:** 1 Graduate School of Science, Osaka University, 1-1 Machikaneyama-cho, Toyonaka 560-0043, Osaka, Japan; 2 Center for Ecological Research, Kyoto University, Otsu 520-2133, Shiga, Japan

**Keywords:** photoperiodism, photoreceptor, circadian clock, Bolwig organ, pigment-dispersing factor, pupal diapause, lateral neuron, Bolwig neuron

## Abstract

**Simple Summary:**

The flesh fly *Sarcophaga similis* shows a clear photoperiodic response, in which its larvae enter pupal diapause as a response to short days, for seasonal adaptation. Although sensitive wavelengths for photoperiodic photoreception have been demonstrated, the photoreceptor organ remains unclear. The aim of this study was to identify the Bolwig organ, a known fly larval-photoreceptor, in *S. similis* and to determine whether the Bolwig organ is the photoperiodic photoreceptor. The Bolwig organ, consisting of more than 30 cells, was located as a tiny spherical body attached to the internal skeleton in the anterior region of *S. similis* larvae. Bolwig-organ neurons extend fibres to terminate near putative circadian-clock neurons in the brain. Surgical ablation of the Bolwig organ cancelled photoperiodic responses, and the incidence of diapause after the surgery was similar to that of intact larvae reared under constant darkness. This suggests that the Bolwig organ partially contributes to photoperiodic photoreception, and other photoreceptors may also be involved. This finding opens the door for the investigation of the neural mechanisms of insect photoperiodism using the simple larval brain of *S. similis*.

**Abstract:**

Flesh-fly *Sarcophaga similis* larvae exhibit a photoperiodic response, in which short days induce pupal diapause for seasonal adaptation. Although the spectral sensitivity of photoperiodic photoreception is known, the photoreceptor organ remains unclear. We morphologically identified the Bolwig organ, a larval-photoreceptor identified in several other fly species, and examined the effects of its removal on the photoperiodic response in *S. similis*. Backfill-staining and embryonic-lethal-abnormal-vision (ELAV) immunohistochemical-staining identified ~34 and 38 cells, respectively, in a spherical body at the ocular depression of the cephalopharyngeal skeleton, suggesting that the spherical body is the Bolwig organ in *S. similis*. Forward-fill and immunohistochemistry revealed that Bolwig-organ neurons terminate in the vicinity of the dendritic fibres of pigment-dispersing factor-immunoreactive and potential circadian-clock neurons in the brain. After surgical removal of the Bolwig-organ regions, diapause incidence was not significantly different between short and long days, and was similar to that in the insects with an intact organ, under constant darkness. However, diapause incidence was not significantly different between the control and Bolwig-organ-removed insects for each photoperiod. These results suggest that the Bolwig organ contributes partially to photoperiodic photoreception, and that other photoreceptors may also be involved.

## 1. Introduction

Photoperiodism is the property of organisms to respond to the day- or night-length of the 24 h-day. The photoperiod conveys seasonal changes with higher accuracy than other physical factors such as temperature. Through photoperiodic responses, many insects prepare for hardiness for seasonal adaptation before the arrival of severe environmental conditions [1]. The physiological mechanisms underlying insect photoperiodic responses involve photoreception, measuring day-length (time measurement), counting the number of short or long days, and endocrine control of physiological states such as diapause [2,3]. Although these physiological constituents have been examined in a variety of species using different methods, the neuronal pathways that convey photoperiodic signals from photoreceptors to the brain remain largely unknown. 

Different circadian-clock-based models have been proposed for time-measurement mechanisms, originating from Bünning’s hypothesis [4,5]. The external-coincidence model is a sophisticated model, exemplified in the flesh flies *Sarcophaga argyrostoma* and *Sarcophaga similis* [6,7]. In this model, day-length is determined, based on the presence or absence of light during the photoinducible phase (φi) in the late scotophase, which is set by the circadian clock entraining to environmental light- and dark-cycles [8]. Therefore, light has a dual role: entrainment of the circadian clock to environmental light/dark cycles and a direct inductive role during φi, which is either illuminated to cause long-day (LD) responses or not illuminated to cause short-day (SD) responses [3,8]. Consequently, two distinct photopigments are suggested to underlie the photoreception in the time-measurement system [8]. To access the cellular components of the time-measurement system, it is important to examine the anatomical pathway of the photoperiodic photoreceptor organs to the brain neurons. Considering the involvement of circadian-clock genes in photoperiodic responses in many insect species [5,9,10], circadian-clock cells appear to be constituents of the time-measurement system in the brain. However, no studies have demonstrated connections between photoperiodic photoreceptors and the circadian-clock cells.

*S. similis* exhibits a photoperiodic response in which SD conditions during its embryonic or larval stages promote pupae to enter diapause [11]. The external-coincidence model applies well to the photoperiodic response in *S. similis* larvae, and the involvement of different types of photoreceptors for circadian entrainment and φi-light exposure has been suggested [7]. Wavelengths of 470 nm or shorter are effective for entrainment of the circadian clock, whereas a broad-wavelength range of 395 to 660 nm is effective for φi-light exposure. Together with the small number of neurons and the simple structure of the larval brain [12], *S. similis* larvae provide an excellent model to investigate the photoperiodic mechanism through photoperiodic photoreception and the circadian-clock system.

The Bolwig organ (BO), known as the larval eye for the entrainment of circadian rhythms and for light avoidance in *Drosophila melanogaster* [13,14], was first described by Niels Bolwig [15] in the house fly *Musca domestica* as larval visual organs located in the anterior small cavity of the cephalopharyngeal skeleton (CPS). The organ was later discovered in several cyclorrhaphous dipterans [16,17,18]. In *D. melanogaster*, the BO resides in a pocket-like structure of the CPS, in which two classes of neurons, one expressing *rhodopsin 5* and the other *rhodopsin 6*, are located [19,20,21,22]. The spectral sensitivities of rhodopsin 5 and 6 were 437 and 508 nm, respectively [23]. In the blow fly *Calliphora vicina*, extracellular recordings from the Bolwig nerve (BN) of the third-instar larvae found that the BO is sensitive to white and green light, with the highest sensitivity at 527 nm [17]. Considering the effective wavelengths from 395 to 660 nm for the photoperiodic response at φi, the BO may be a potential photoperiodic photoreceptor in *S. similis* larvae [7,17,23].

The long fibres of BO cells of *D. melanogaster* extend in the BN along the antennal and eye disks to reach the brain, where circadian-clock cells reside [19,24,25]. Terminal endings of BO cells are in the immediate vicinity of a dendritic region of clock cells called lateral neurons (LNs) [14]. The BO is suggested to transmit light signals to the LNs to entrain the molecular clock [13]. If the anatomical properties and spectral sensitivity of the BO in *D. melanogaster* are comparable in *S. similis*, the BO and LNs are strong candidates for neural components constituting a simple photoreceptor and time-measurement system in *S. similis*.

In the present study, we anatomically describe the BO in *S. similis* larvae, and examine its role in the photoperiodic response controlling pupal diapause by surgical ablation. Furthermore, we show that BN terminals are in close proximity to pigment-dispersing factor (PDF)-immunoreactive neurons using immunohistochemistry (IHC) and the forward-fill technique, and discuss the potential role of larval BO in photoperiodic photoreceptors.

## 2. Materials and Methods

### 2.1. Insects

*S. similis* (Diptera: Sarcophagidae) larvae of the second to fourteenth generations from adults collected from 2017 to 2021 at the Toyonaka Campus of Osaka University (34.80° N, 135.45° E) were used. Insect-rearing methods were adopted from a previous study [7]. Fly cultures were maintained at 20.0 ± 1.5 °C under LD conditions of 16 h light (~5.3 Wm^2^) and 8 h darkness (16L8D). Fifteen to thirty adult flies kept in a circular plastic container (diameter 15.0 cm, height 9.0 cm) were provided with water and sucrose ad libitum. Within a week of adult emergence, a piece of chicken liver (the first liver piece) was provided as a protein source for the development of female ovaries. Fourteen days later, the second liver piece was provided for larviposition. Liver pieces were given at an interval of two weeks until death, and each liver feeding caused larviposition. Typically, three or four liver pieces had to be provided. Larvae on the day of larviposition (day 0) were collected on a large piece of liver placed on top of wood chips, in which the third instar larvae would wander on days 5–7 in search of a place to pupariate. All larval experiments were performed during their wandering stage.

### 2.2. Forward-Fill and Backfill Staining

**Figure 1:**
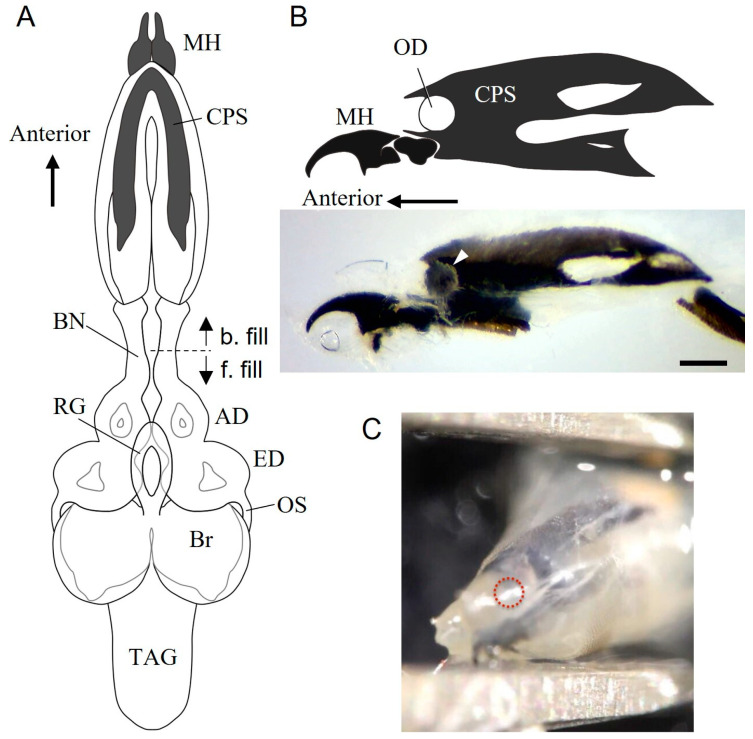
Structure of internal organs in the anterior part of *Sarcophaga similis* larvae. (**A**) Schematic illustration of the mouth hook (MH), cephalopharyngeal skeleton (CPS), antennal disk (AD), eye disk (ED), ring gland (RG), brain (Br) and thoracicoabdominal ganglion (TAG) in a dorsal view. The Bolwig nerve (BN) was cut at the dotted line to fill dye backward (b. fill) or forward (f. fill). (**B**) Diagram and photo of the MH and CPS in a sagittal view. A spherical body is present in the ocular depression (OD, arrowhead). (**C**) Anterior oblique view of the left MH and CPS. The red dotted circle shows the region of the OD into which a tungsten needle was inserted for ablation. Scale bar = 200 µm.

The BN of a wandering larva was dorsally exposed and cut between the antennal disk and CPS (Figure 1A). A cut end connected to the brain through the antennal disk (forward-fill) or that to the CPS region (backfill) was placed in an opening tip (approximately 0.1 mm) of a sharpened glass capillary (1 mm in diameter) containing dextran, tetramethylrhodamine and biotin-conjugated 3000 MW, Lysine Fixable (130 mM, D7162, Molecular Probes, Eugene, OR, USA) or neurobiotin (150 mM, SP-1120, Vector Laboratories, Newark, CA, USA). This preparation (larva with the dye-filled capillary) was kept in a moist chamber for 2–5 h at 25–30 °C, or overnight at 4 °C. The dye-filled brain or CPS region was removed and fixed in 4% paraformaldehyde overnight, at 4 °C. After neurobiotin filling, the organs were incubated with avidin-biotin complex (1:100, the Vectastain^®^ ABC kit, Vector Laboratories) for 1 h at 25–30 °C, then with Alexa Fluor 488-conjugated streptavidin (1:200, S-11223, Invitrogen, Carlsbad, CA, USA) for overnight, at 4 °C. The brain or CPS region was dehydrated using an ethanol series, and cleared using methyl salicylate.

### 2.3. Immunohistochemistry

The brain or the CPS region of a wandering larva was dissected and fixed in 4% paraformaldehyde overnight, at 4 °C. The specimens were washed three times with 0.1 M phosphate buffered saline with Tween 20 (PBST, pH = 7.45, 0.05% Tween), blocked for 1 h using 5% normal donkey serum (NDS, IHR-8135, ImmunoBioScience Corp., Mukilteo, WA, USA) in PBST, and placed in a mixture of 0.1 M PBST with 5% NDS and primary antibody for three nights, at 4 °C. On the fourth day, they were washed with PBST and placed in a solution of 0.1 M PBST with 5% NDS and secondary antibody overnight, at 4 °C. The primary and secondary antibodies and their dilutions are listed in Table 1. Two types of anti-PDF antibody were used in this study. No clear staining-differences were produced between the two antibodies. The specimens were dehydrated using an ethanol series, and cleared using methyl salicylate. For double staining, forward-fill or backfill staining was conducted, as described in Section 2.2, followed by IHC.

### 2.4. Microscopy

**Figure 2:**
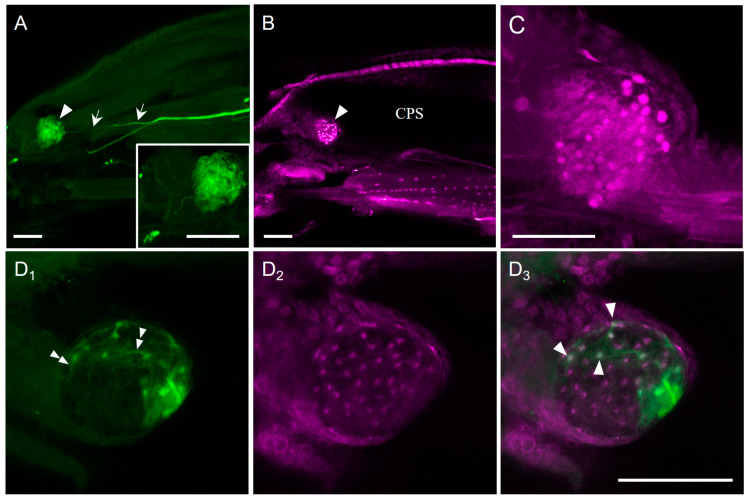
Photomicrographs showing the Bolwig organ (BO) at the ocular depression of the anterior region of the cephalopharyngeal skeleton (CPS) in a sagittal view of *Sarcophaga similis*, upper to dorsal, left to anterior. (**A**) Backfill-staining with neurobiotin from the cut end of the Bolwig nerve (see Figure 1A). A bifurcated thin fibre (arrows) reaches to the ocular depression (arrowhead). Inset shows enlarged view of the ocular depression. A stack of 107 confocal sections with a pixel size of 1.66 μm and voxel-depth 0.5 μm. (**B**,**C**) Many ELAV-immunoreactive cells were found in the ocular depression (arrowhead). A single confocal section with a pixel size of 1.42 μm for (**B**) and a stack of 41 confocal sections with a pixel size of 0.21 μm with voxel-depth 1.0 μm for (**C**,**D**). Double staining of the BO by backfill (green, (**D_1_**)) and ELAV immunohistochemistry (magenta, (**D_2_**)). Thin fibres were seen during backfill-staining (double arrowheads). (**D_3_**) is a merged image. Single arrowheads indicate double-labelled cells. A stack of 68 confocal sections with a pixel size of 0.83 μm and voxel-depth 1.0 μm. Scale bar = 100 µm.

**Figure 3:**
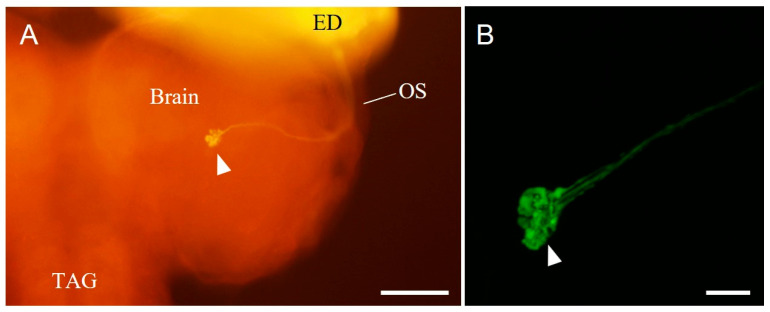
Photomicrographs of neuron terminals stained by forward-fills from the cut end of the Bolwig nerve (see Figure 1A) in the larval brain of *Sarcophaga similis* (a dorsal view, upper to anterior). (**A**) Fluorescent photomicrograph showing dextran rhodamine backfilled-fibres. Stained fibres run through the eye disk (ED) and optic stalk (OS) to reach the middle of the brain hemisphere (arrowhead). (**B**) Large swelling is obvious at the terminal ending (arrowhead, neurobiotin-staining). A stack of 184 confocal sections with a pixel size of 0.13 μm and voxel-depth 0.4 μm. TAG, thoracicoabdominal ganglion. Scale bar = 100 µm in (**A**), 20 µm in (**B**).

**Figure 4:**
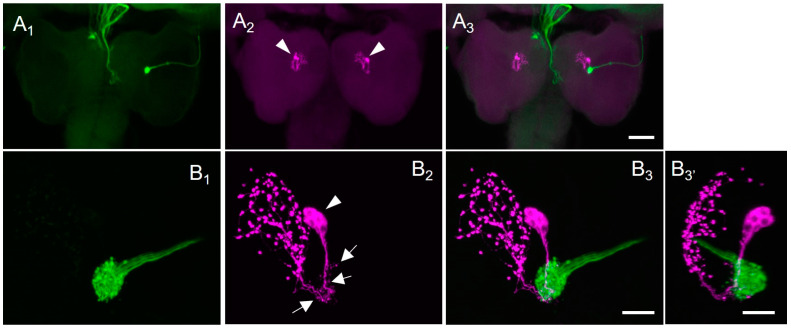
Photomicrographs of the larval brain doubly stained with forward-fill ((**A_1_**,**B_1_**), green) and pigment-dispersing factor (PDF) immunohistochemistry ((**A_2_**,**B_2_**), magenta, mouse anti-*Drosophila* PDF antibody) in *Sarcophaga similis*. (**A_3_**,**B_3_**) are merged images of (**A_1_**) with (**A_2_**) and (**B_1_**) with (**B_2_**), respectively. (**B_3′_**) is an image of (**B_3_**) at a different angle. (**A**) A pair of clusters (arrowheads) of PDF-immunoreactive neurons were present in the brain. A stack of 52 confocal sections with a pixel size of 1.66 μm and voxel-depth 3.16 μm. (**B**) Four PDF-immunoreactive cells (arrowhead) and small thin fibres (arrows) are distinguished. In both views represented by (**B_3_**,**B_3′_**), forward-filled terminals from the Bolwig nerve are in the close vicinity of PDF-immunoreactive fibres. A stack of 237 confocal sections with a pixel size of 0.26 μm and voxel-depth 0.5 μm. Scale bar = 100 µm in (**A**), 20 µm in (**B**).

Fluorescent images (Figure 2, Figure 3B and Figure 4) were captured using a confocal laser scanning microscope (LSM 710, Carl Zeiss, Jena, Germany) with objective lenses (Plan-Apochromat 10×/0.45, EC Plan-Neofuar 20×/0.50, Plan-Apochromat 40×/0.95, and Plan-Apochromat 63 × 1.4 oil, Carl Zeiss). Alexa Fluor 488, TRITC, and Alexa Fluor 647 were excited using an argon laser (488 nm), diode-pumped solid-state laser (561 nm), and red HeNe laser (633 nm), respectively. The emission light was detected using appropriate filter sets for the respective fluorescent dyes. Optical sections were reconstructed using an image-processing software (ZEN blue, Version 3.5.093.00006, Carl Zeiss). Figure 3A shows the image captured using an epifluorescence microscope (BX-51, Olympus, Tokyo, Japan).

### 2.5. Assessment of Photoperiodism

Adult flies were kept under SD conditions (12L12D) from the day the first liver piece was given. Day 0 larvae deposited on the day of administering the second liver piece were collected and reared until the third instar, when they ceased feeding and started wandering (days 5–7). They were divided into four groups and subjected to water treatment, in which the larvae were confined in 60 mm laboratory dishes containing wood chips with approximately 4 mL of water, to extend the wandering period [7]. Each group under water-treatment was transferred to SD, LD, constant-light (LL), or constant-dark (DD) conditions within one day of the onset of wandering, so that the larvae would experience their respective conditions for 5 days. On the sixth day of water treatment, the larvae were released into dry woodchips for pupariation. Nondiapause flies emerged 14–16 days after pupariation. Eclosed adults were recorded as nondiapause, and pupae were examined for survival 14 to 21 days after pupariation. Pupae with no sign of pigmentation (white) in the head were regarded as having entered diapause. The numbers of nondiapause, diapause, and dead flies were recorded.

### 2.6. BO Ablation

The BO at the ocular depression (Figure 1B) [26] of the anterior part of the CPS was impaired at the onset of wandering (days 5–7) under SD conditions. Larvae were placed on ice for cold anaesthesia at least 20 min prior to ablation. As the location of the ocular depression was found through the translucent cuticle (Figure 1C), a sharpened insect pin (<0.05 mm in diameter) was used to poke a hole bilaterally on the cuticle of the anterior end, and a tungsten needle (approximately 0.03 mm diameter) was inserted through the hole to reach the ocular depression and damage it (Figure 1C). The BO was removed bilaterally. For the sham operation, larvae were subjected to anaesthetisation and to the creation of cuticular holes, without damaging the ocular depression. Larvae of the BO-damaged group, along with those in the sham and intact groups, were reared under water treatment for five days in SD or LD conditions, and then pupariated under dry conditions with the same photoperiod. All pupae were maintained under SD conditions to examine diapause or nondiapause destiny, as described above. In flies emerging from nondiapause pupae, the presence or absence of the eyelet, a photoreceptor derived from larval BO [24], was examined after two days or more of adult emergence. In *D. melanogaster* and the blow fly *Protophormia terraenovae*, the eyelet resides at the posterior surface of the optic lobe between the retina and lamina [27,28]. To examine the eyelet, the head of the fly was opened to expose the posterior face of the optic lobe.

### 2.7. Statistical Testing

For multiple comparisons of diapause incidences in BO ablation experiments, a Tukey-type multiple-comparison test for proportions was conducted using the R software ver. 4.1.0 (http://cran.r-project.org) with an additional package (“http://aoki2.si.gunma-u.ac.jp/R/src/p_multi_comp.R”, encoding = “euc-jp” ((accessed on 9 January 2023)).

## 3. Results

### 3.1. Internal Structure of the Anterior End of Wandering Larvae

At the anterior end of the larva, the dark-coloured mouth hooks and CPSs covered with many pharyngeal muscles were found to be similar to those in *C. vicina* (Figure 1A) [29]. The CPS region was posteriorly connected to the eye-antennal imaginal discs, forming a tube-like structure. From the eye disk, the optic stalk [30] extended to enter the brain in the anterior lateral region (Figure 1A). Several thin nerves deviated anteriorly from the antennal disc, although they were difficult to follow.

### 3.2. Bolwig Organ in S. similis Larvae

When adjacent fat bodies and muscle layers were removed from the CPS, a spherical body was observed at the pocket-like ocular depression at the anterior edge of the CPS (Figure 1B). When the nerve between the CPS and antennal disk was cut off and backfilled towards the CPS, a stained fibre was observed, reaching the CPS ocular depression where the spherical body was labelled (Figure 2A). The diameter of the stained body was 111.1 ± 8.6 μm (average ± S.D., N = 3). The cell number per body was 34.0 ± 1.4 (N = 2).

In *D. melanogaster*, embryonic lethal abnormal vision (ELAV), a neuronal protein required for the development and maintenance of the nervous system, is expressed in BO cells [31]. In *S. similis* larvae, ELAV immunoreactivity was found in the CPS ocular depression, where 38.3 ± 7.4 cells (N = 9) were labelled (Figure 2B,C). The diameter of the ELAV-immunoreactive cells was 5.9 ± 0.9 µm (N = 9, n = 90). The maximum diameter of the entire spherical region containing ELAV-immunoreactive cells was 94.5 ± 14.5 μm (N = 8). In double-staining preparations with anti-ELAV antibody, backfills labelled a smaller number of cells, due to weak staining (Figure 2D_1_). Thin fibres arising from the dot-like cells were also observed in the spherical body (Figure 2D_1_). These may be retinal fibres running to the BN. Out of forty-one ELAV-immunoreactive cells, nine were also stained with backfills (Figure 2D). Because of ELAV-immunoreactivity and a homologous anatomical position to *M. domestica* [15], the spherical body and cells at the CPS ocular depression in *S. similis* appeared to be BO and BO neurons, respectively.

### 3.3. Terminals of the Bolwig-Organ Neurons in the Brain

The forward-fill from the cut end of the BN stained a nerve bundle projecting through the antennal and eye disks to enter the brain via the optic stalk (Figure 3A). A large terminal swelling was found ipsilaterally in the medial region of the hemisphere, and many dot-like structures were observed in the swelling (Figure 3B). The size of the total terminal swelling was 26.6 ± 2.1 µm on the anteroposterior axis, 18.0 ± 1.0 µm on the lateral axis, and 39.8 ± 7.1 µm on the dorsoventral axis (N = 3). These terminals are possibly derived from BO neurons.

After unilateral forward-filling of the BN (Figure 4A_1_,B_1_), PDF immunohistochemistry was performed (Figure 4A_2_,B_2_). A pair of PDF-immunoreactive cell clusters was found in the brain (Figure 4A_2_). The PDF-immunoreactive cells had ventrally protruded neurites and bore weakly stained, fine branches in the ventral regions (Figure 4B_2_). These fibres appear to be in a dendritic region. Dorsally distinct varicose fibres were stained in the protocerebrum (Figure 4B_2_). Single PDF immunohistochemistry revealed that the number of PDF-immunoreactive cells per hemisphere was 4.3 ± 1.2 (N = 8), and their cell diameter was 8.4 ± 2.2 μm (N = 8, n = 32). Double staining with PDF immunohistochemistry and forward-fills revealed that the swelling terminals from the BN were in the proximity of the dendritic region of PDF-immunoreactive neurons (Figure 4A_3_,B_3_,B_3′_).

### 3.4. Effects of BO Ablation on Photoperiodic Response

**Figure 5:**
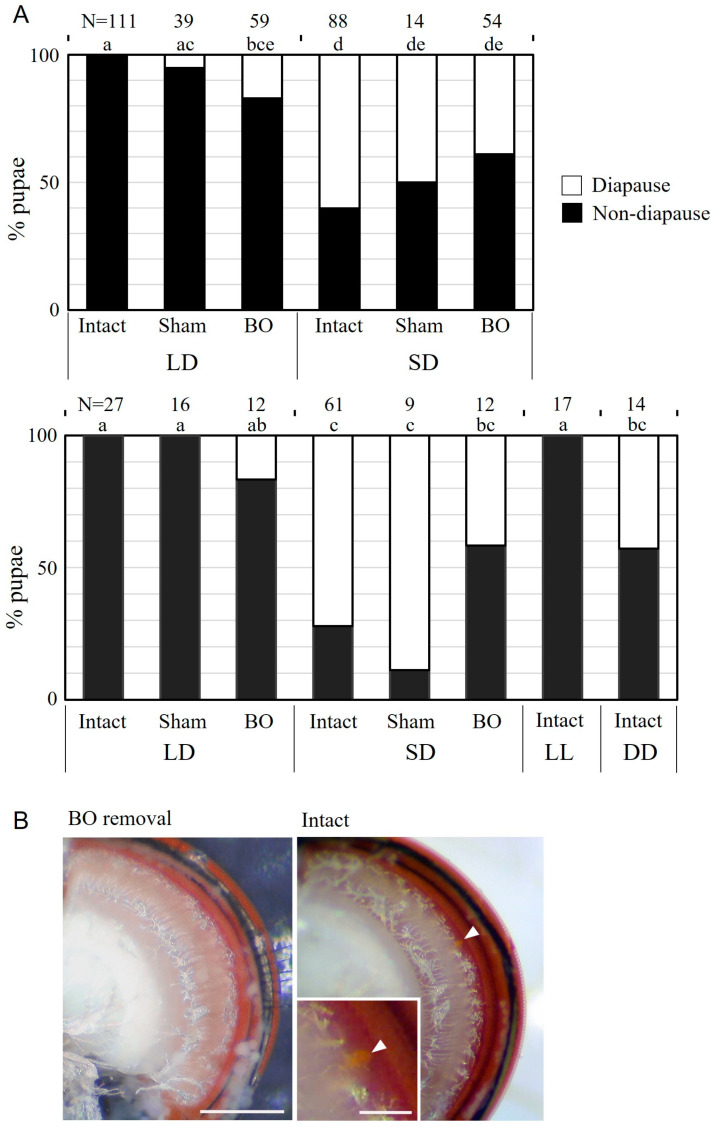
Effects of the Bolwig-organ removal on the photoperiodic response controlling pupal diapause in *Sarcophaga similis* larvae. (**A**) Two series of experiments (upper, the 2020 culture; lower, the 2021 culture). As controls, intact and sham-operated groups were prepared. In the Bolwig-organ-removal group (BO), no significant difference was detected between insects under long-day (LD) and short-day (SD) conditions. Columns with different letters show significant difference in their diapause incidence (Tukey-type multiple comparisons for proportions, *p* < 0.05). (**B**) Stereomicroscopic photos of posterior face of the optic lobe in the adult fly. A small eyelet (orange colour, arrowheads) is located between the retina (red region) and neuropil region (white region) of an intact fly. After BO removal, the eyelet was absent. Scale bar = 100 µm.

We conducted BO-ablation experiments twice, using different fly cultures. In the culture from flies collected in 2020, the photoperiodic response was not obvious, although diapause incidence in the insects of the intact group was significantly higher under SD than under LD conditions (Figure 5A). The sham operation did not cause significant effects in either photoperiodic condition. When the BO region was bilaterally ablated, diapause incidence tended to increase slightly under LD conditions, compared with that in the intact group (Figure 5A). Under SD conditions, the diapause incidence did not differ among the insects of intact, sham, or BO-ablation groups (Figure 5A). In the fly culture from the strain collected in 2021, the photoperiodic responses in insects of the control groups were clearer, but diapause incidence was not significantly different among the insects of intact, sham, and BO-ablation groups under both SD and LD conditions. However, in both the fly cultures, diapause incidence did not differ between the insects under SD and LD conditions in the BO-ablated group (Figure 5A). In the intact group, the diapause incidences under LL and DD conditions were significantly different, and the diapause incidences after BO ablation under both SD and LD conditions were not significantly different from those in the insects of the intact-DD group (Figure 5A).

To evaluate the success of BO ablation, we examined whether the eyelets in the emerging adults disappeared. In intact flies, the orange structure of the eyelet was located on the posterior face of the optic lobe, close to the retina (Figure 5B). Among the 34 flies that emerged from the culture initiated in 2020 and were successfully examined for BO ablation, 88.2% flies lost the eyelets on both sides and 11.8% flies lost the eyelet on a single side, with no remaining flies possessing eyelets bilaterally. In the 2021 culture, bilateral eyelets disappeared in 92.3% (N = 13) and one eyelet remained in 7.7%. The absence of eyelet after BO ablation is shown in Figure 5B.

## 4. Discussion

### 4.1. Bolwig Organ and Its Neuronal Terminals in the Brain of S. similis

*D. melanogaster* expresses ELAV, a neuronal protein necessary for the development and maintenance of the nervous system, in BO neurons [31,32]. We also found ELAV-immunoreactive cells at the ocular depression of the CPS in *S. similis*. Backfills from the BN also revealed a similar number of cells in the same region. Furthermore, co-localisation was observed between the backfill and ELAV-immunohistochemical labelling. These results strongly suggest that the ELAV-immunoreactive or backfill-stained cells from the BN are the somata of BO neurons in *S. similis*. The number of BO neurons was approximately three times higher in *S. similis* than in *D. melanogaster*. While *D. melanogaster* contains ~12 cells, ~38 cells were found in the ocular depression of *S. similis* [20,33]. The anatomy of the head region and the position of the BO have been analysed by making semi-thin sections of different dipteran species cited by Melzer and Paulus [16]. In the BO, 32 cells have been identified in *Lonchoptera* spec, 44 in *Volucella bombylans*, approximately 30 in *Atherix ibis*, and 33 in *M. domestica*. The large number of BO neurons in other fly species than *D. melanogaster*, including *S. similis,* may suggest a developed larval-photoreceptor system compared with that in *D. melanogaster*, and its multiple roles, including photoperiodic photoreception, depending on the species.

In *D. melanogaster*, developmental studies have shown that the BO is composed of four photoreceptors expressing blue-sensitive rhodopsin 5 and eight photoreceptors expressing green-sensitive rhodopsin 6 [20]. These two types of photoreceptor cells are functionally different. Although both types of photoreceptors mediate the photic entrainment of the circadian-clock system in the brain, only the rhodopsin-5 photoreceptor is essential for light-avoidance behaviour [21]. To analyse the cellular composition of the BO, immunohistochemical or in situ hybridisation studies for rhodopsins in *S. similis* must be carried out in the future.

Afferent fibres from the BN in *S. similis* enter the brain, to terminate in the vicinity of the PDF-immunoreactive fibres, as in *D. melanogaster* [19,34]. In *S. similis* larvae, PERIOD-immunoreactive LNs were found in the middle of the brain hemisphere [35]. Considering that PDF and PERIOD are colocalised in the LNs in the larvae and adults of *D. melanogaster* as well as in the adults of *P. terraenovae* [34,36,37], PDF-immunoreactive neurons in *S. similis* larvae may correspond to these LNs. Interestingly, temporal changes in the number of PERIOD-immunoreactive LNs have been observed in the *S. similis* larval brain, in which the temporal patterns differed between larvae exposed to SD and LD conditions [35]. This suggests that the *S. similis* larval LNs receive photoperiodic signals.

Nerve terminals from the BO and ventral branches of the PDF-immunoreactive neurons appeared to connect morphologically. Because of the weak immunoreactivity and different branching patterns from the dorsal projections, the PDF-immunoreactive ventral fibres in *S. similis* larvae (Figure 4B_2_) can be considered dendritic regions, as reported in *D. melanogaster* [38]. In *D. melanogaster*, the absence of the BO or inhibition of afferent-BN activity results in alterations in dendritic arborisation of the pigment-dispersing hormone PDH- (a PDF orthologue peptide) immunoreactive LNs [19]. As immunostaining intensity against the circadian-clock proteins of PERIOD and TIMELESS exhibits daily changes at the cellular level under light/dark and constant-dark conditions, the PDH-immunoreactive LNs are considered larval circadian-clock cells [34]. Light exposure to the BO caused calcium activity in the lateral neurons, and changes in environmental lighting conditions induced substantial structural plasticity in the dendritic arbores of the LNs in *D. melanogaster* [38]. These studies clearly indicate the physiological connections between BO photoreceptors and LNs in the larval brain. Morphological observations from the present study, together with the related findings in *D. melanogaster* suggest that BO neurons in *S. similis* interact functionally with the PDF-immunoreactive LNs in the larval brain. If this is the case, the pathway from BO neurons to the circadian-clock PDF-immunoreactive LNs is a potential candidate route for conveying photoperiodic information. The responsiveness of LNs to day-length also supports this idea [35].

### 4.2. Roles of the BO in Photoperiodism

To investigate the role of the BO in photoperiodic response, we surgically ablated the BO. Although not all flies were examined, the eyelet was not found in approximately 90% of adult flies after BO removal at the larval stage, suggesting that the surgery was mostly successful. The eyelet is an extraretinal photoreceptor organ in adult flies, developmentally derived from the larval BO [24]. Diapause incidences in insects that had their BOs removed showed an intermediate response between SD-control insects and LD-control insects, but they were not significantly different from those in control flies in each photoperiodic condition. This suggests the possibility that the BO was not involved in the photoperiodic response. However, photoperiodic difference in the incidence of pupal diapause disappeared among the BO-ablated groups. These results suggest that the BO contributes, at least partially, to photoperiodic photoreception, and other photoreceptors may also be involved.

Cryptochrome is known to participate in circadian photoreception in *D. melanogaster* larvae [13]. Two types of clock neurons, LNs and dorsal neuron 1, in the larval brain express cryptochrome [39]. Another group of photosensitive cells has also been reported as Class IV dendritic-arborisation neurons on the body wall of *D. melanogaster* larvae [40]. However, the responsive wavelengths of these photoreceptors do not cover the entire range of wavelengths required for the photoperiodic response of *S. similis* [7]. Cryptochrome in *D. melanogaster* have peak wavelength-sensitivity at 450 nm, with defined shoulders around 420 and 480 nm, but no longer than 500 nm [41]. Class IV dendritic-arborisation neurons are responsive to short-wavelength light from ultraviolet (365 nm) to blue (470 nm) light but not to longer wavelengths in the range from green (525 nm) to red (620 nm) [40]. For the photoperiodic response controlling pupal diapause in *S. similis* larvae, a wavelength of 470 nm or shorter is effective for entrainment of the circadian clock, and a broad-wavelength range from 395 to 660 nm is required to switch from SD to LD responses at φi under the external-coincidence model [7]. Cryptochrome and Class IV dendritic-arborisation neurons may contribute to photic entrainment of the circadian clock involved in the photoperiodic response; however, they cannot accomplish the response alone. For the reception of long-wavelength light, rhodopsin 5 or 6 in the BO may work together with rhodopsin molecules in other organs for photoperiodic photoreception.

Diapause incidences in BO-removed insects under SD and LD conditions were not significantly different from diapause incidence in intact insects under DD. In intact insects, diapause incidence under DD was approximately 45%, and this value was intermediate between those under SD and LD conditions. This supports the results of a previous study in *S. similis* larvae, that the response to DD conditions is different from that to SD or to LD conditions [42]. A similar diapause incidence between insects without the BO and intact insects with the BO under DD conditions suggests that the absence of the BO may provide no light information to the brain, causing a similar response to DD perception. This also supports the possibility that BO acts as a photoperiodic photoreceptor.

## Figures and Tables

**Table 1:** Antibodies used for immunohistochemistry.

Target Peptide or Protein	Primary Antibody	Secondary Antibody
Pigment-dispersing factor (PDF)	Rabbit anti-*Gryllus* PDF antibody for single immunohistochemistry (RRID:AB_2916037, from Dr. Tomioka) 1:1000	Donkey anti-Rabbit IgG (H + L) Secondary Antibody, TRITC (A-16028, Invitrogen) 1:1000
	Mouse anti-*Drosophila* PDF antibody for double staining with forward-fill (RRID:AB_760350, DSHB PDF C7) 1:100	Goat anti-Mouse IgG (H + L) Cross-Adsorbed Secondary Antibody, Alexa Fluor™ 647 (A-21235, Invitrogen) 1:1000
Embryonic lethal abnormal vision (ELAV)	Rat anti-*Drosophila* ELAV antibody (RRID:AB_528218, DSHB Rat-ELAV-7E8A10 anti-ELAV) 1:200	Goat anti-Rat IgG (H + L) Cross-Adsorbed Secondary Antibody, Alexa Fluor™ 647 (A-21247, Invitrogen) 1:500

## Data Availability

All relevant data are within the paper.

## References

[1] Tauber M.J., Tauber C.A., Masaki S. (1986). Seasonal Adaptations of Insects.

[2] Saunders D.S. (2002). Insect Clocks.

[3] Saunders D.S. (2021). Insect photoperiodism: Bünning’s hypothesis, the history and development of an idea. Eur. J. Entomol..

[4] Bünning E. (1936). Die endogene Tagesrhythmik als Grundlage der Photoperiodischen Reaktion. Ber. Dtsch. Bot. Ges..

[5] Saunders D.S. (2020). Dormancy, Diapause, and the role of the circadian system in insect photoperiodism. Annu. Rev. Entomol..

[6] Saunders D.S. (1979). External coincidence and the photoinducible phase in the *Sarcophaga* photoperiodic clock. J. Comp. Physiol..

[7] Goto S.G., Numata H. (2009). Possible involvement of distinct photoreceptors in the photoperiodic induction of diapause in the flesh fly *Sarcophaga similis*. J. Insect Physiol..

[8] Pittendrigh C.S., Minis D.H. (1964). The entrainment of circadian oscillations by light and their role as photoperiodic clocks. Am. Nat..

[9] Meuti M.E., Denlinger D.L. (2013). Evolutionary links between circadian clocks and photoperiodic diapause in insects. Integr. Comp. Boil..

[10] Goto S.G. (2022). Photoperiodic time measurement, photoreception, and circadian clocks in insect photoperiodism. Appl. Entomol. Zool..

[11] Tanaka M., Tachibana S.I., Numata H. (2008). Sensitive stages for photoperiodic induction of pupal diapause in the flesh fly *Sarcophaga similis*. Appl. Entomol. Zool..

[12] Raji J.I., Potter C.I. (2021). The number of neurons in *Drosophila* and mosquito brains. PLoS ONE.

[13] Malpel S., Klarsfeld A., Rouyer F. (2004). Circadian synchronization and rhythmicity in larval photoperception-defective mutants of *Drosophila*. J. Biol. Rhythms.

[14] Mazzoni E.O., Desplan C., Blau J. (2005). Circadian pacemaker neurons transmit and modulate visual information to control a rapid behavioral response. Neuron.

[15] Bolwig N. (1946). Senses and sense organs of the anterior end of the house fly larvae. Vidensk. Medd. Fra Dan. Nat. Foren. Kbh..

[16] Melzer v.R.R., Paulus H. (1989). Evolutionary pathways to the larval eyes of insects-higher dipteran stemmata and the evolutionary development of Bolwig’s organ. Z. Zool. Syst. Evol..

[17] Hinnemann A., Niederegger S., Hanslik U., Heinzel H.-G., Spieß R. (2010). See the light: Electrophysiological characterization of the Bolwig organ’s light response of *Calliphora vicina* 3rd instar larvae. J. Insect Physiol..

[18] Schmucker D., Taubert H., Jäckle H. (1992). Formation of the *Drosophila* larval photoreceptor organ and its neuronal differentiation require continuous Krüppel gene activity. Neuron.

[19] Malpel S., Klarsfeld A., Rouyer F. (2002). Larval optic nerve and adult extra-retinal photoreceptors sequentially associate with clock neurons during *Drosophila* brain development. Development.

[20] Sprecher S.G., Pichaud F., Desplan C. (2007). Adult and larval photoreceptors use different mechanisms to specify the same rhodopsin fates. Genes Dev..

[21] Keene A.C., Mazzoni E.O., Zhen J., Younger M.E., Yamaguchi S., Blau J., Desplan C., Sprecher S.G. (2011). Distinct visual pathways mediate *Drosophila* larval light avoidance and circadian clock entrainment. J. Neurosci..

[22] Sprecher S.G., Desplan C. (2008). Switch of rhodopsin expression in terminally differentiated *Drosophila* sensory neurons. Nature.

[23] Salcedo E., Huber A., Henrich S., Chadwell L.V., Chou W.-H., Paulsen R., Britt S.G. (1999). Blue- and green-absorbing visual pigments of *Drosophila*: Ectopic expression and physiological characterization of the R8 photoreceptor cell-specific Rh5 and Rh6 rhodopsins. J. Neurosci..

[24] Helfrich-Förster C., Edwards T., Yasuyama K., Wisotzki B., Schneuwly S., Stanewsky R., Meinertzhagen I.A., Hofbauer A. (2002). The extraretinal eyelet of *Drosophila*: Development, ultrastructure, and putative circadian function. J. Neurosci..

[25] Farca-Luna A.J., Sprecher S.G. (2013). Plasticity in the *Drosophila* larval visual system. Front. Cell. Neurosci..

[26] Ruiz-Martinez I., Soler-Cruz M.D., Benitez-Rodriguez R., Perez-Jimenez J.M., Diaz-Lopez M. (1989). Postembryonic development of *Wohlfahrtia magnifica* (Schiner, 1862) (Diptera: Sarcophagidae). J. Parasitol..

[27] Yasuyama K., Meinertzhagen I. (1999). A Extraretinal photoreceptors at the compound eye’s posterior margin in *Drosophila melanogaster*. J. Comp. Neurol..

[28] Yasuyama K., Okada Y., Hamanaka Y., Shiga S. (2006). Synaptic connections between eyelet photoreceptors and pigment dispersing factor-immunoreactive neurons of the blowfly *Protophormia terraenovae*. J. Comp. Neurol..

[29] Schoofs A., Niederegger S., Spieß R. (2009). From behavior to fictive feeding: Anatomy, innervation and activation pattern of pharyngeal muscles of *Calliphora vicina* 3rd instar larvae. J. Insect Physiol..

[30] Meinertzhagen I.A., Young D. (1973). Development of the compound eye and optic lobe of insects. Developmental Neurobiology of Arthropods.

[31] Suzuki T., Saigo K. (2000). Transcriptional regulation of *atonal* required for *Drosophila* larval eye development by concerted action of Eyes absent, Sine oculis and Hedgehog signaling independent of Fused kinase and Cubitus interruptus. Development.

[32] Zhou Q., Yu L., Friedrich M., Pignoni F. (2017). Distinct regulation of *atonal* in a visual organ of *Drosophila*: Organ-specific enhancer and lack of autoregulation in the larval eye. Dev. Biol..

[33] Steller H., Fischbach K.-F., Rubin G.M. (1987). *disconnected*: A locus required for neuronal pathway formation in the visual system of *Drosophila*. Cell.

[34] Kaneko M., Helfrich-Förster C., Hall J.C. (1997). Spatial and temporal expression of the period and timeless genes in the developing nervous system of *Drosophila*: Newly identified pacemaker candidates and novel features of clock gene product cycling. J. Neurosci..

[35] Yamamoto M., Shiga S., Goto S.G. (2017). Distribution of PERIOD-immunoreactive neurons and temporal change of the immunoreactivity under long-day and short-day conditions in the larval brain of the flesh fly *Sarcophaga similis*. Chronobiol. Int..

[36] Helfrich-Förster C. (1995). The period clock gene is expressed in central nervous system neurons which also produce a neuropeptide that reveals the projections of circadian pacemaker cells within the brain of *Drosophila melanogaster*. Proc. Natl. Acad. Sci. USA.

[37] Shiga S., Numata H. (2009). Roles of PER immunoreactive neurons in circadian rhythms and photoperiodism in the blow fly, *Protophormia terraenovae*. J. Exp. Biol..

[38] Yuan Q., Xiang Y., Yan Z., Han C., Jan L.Y., Jan Y.N. (2011). Light-induced structural and functional plasticity in *Drosophila* larval visual system. Science.

[39] Klarsfeld A., Malpel S., Michard-Vanhée C., Picot M., Chélot E., Rouyer F. (2004). Novel features of cryptochrome-mediated photoreception in the brain circadian clock of *Drosophila*. J. Neurosci..

[40] Xiang Y., Yuan Q., Vogt N., Looger L.L., Jan L.Y., Jan Y.N.l. (2010). Light-avoidance-mediating photoreceptors tile the *Drosophila* larval body wall. Nature.

[41] Hoang N., Schleicher E., Kacprzak S., Bouly J.-P., Picot M., Wu W., Berndt A., Wolf E., Bittl R., Ahmad M. (2008). Human and *Drosophila* cryptochromes are light activated by flavin photoreduction in living cells. PLoS Biol..

[42] Goto S.G., Numata H. (2009). Alteration of the pupal diapause program and regulation of larval duration by photoperiod in the flesh fly *Sarcophaga similis* Meade (Diptera: Sarcophagidae). App. Entomol. Zool..

